# Self-Ligating Brackets and Their Impact on Oral Health-Related Quality of Life in Chinese Adolescence Patients: A Longitudinal Prospective Study

**DOI:** 10.1155/2014/352031

**Published:** 2014-08-17

**Authors:** Yu Zhou, MinLing Zheng, Jiaqiang Lin, Yi Wang, Zhen Yu Ni

**Affiliations:** Department of Orthodontics, Hospital of Stomatology, Wenzhou Medical University, No. 113 Xueyuan Xi Road, Wenzhou 325027, China

## Abstract

*Introduction*. Although the associations between orthodontic and oral health-related quality of life (OHRQOL) have been explored, little research has been done to address the influence of brackets type on perceived OHRQOL. The aim of this study was to assess whether the levels of OHRQOL in Chinese adolescence patients were influenced by the type of brackets. *Materials and Methods*. One hundred fifty Chinese orthodontic adolescence patients completed the 14-item Oral Health Impact Profile (OHIP-14, Chinese version) at five distinct intervals: after insertion of the fixed appliance at 1 week (T1), 1 month (T2), 3 months (T3), and 6 months (T4); and after treatment (T5). *Results*. Patients with self-ligating brackets were associated with less pain and discomfort at any intervals compared with conventional brackets, but no significant difference of overall OHIP-14 scores could be found between two groups. Moreover, in both groups, overall scores at T1 and T2 were significantly higher than the scores at any other intervals in both groups. *Conclusions*. The type of orthodontic appliance did not affect oral health-related quality of life in Chinese adolescence patients.

## 1. Introduction

With the medical model transforming from pure biomedical model to the “biological psychological social” medical model, the study of orthodontics is also converting its pattern from the traditional biomedical model [1] to a biopsychosocial perspective [2], and increasing focus has been given to the issues regarding oral health-related quality of life (OHRQOL) [3]. OHRQOL is defined as the absence of negative impacts of oral conditions on social life and a positive sense of dentofacial self-confidence [4].

Self-ligating brackets (SLBs) are not new conceptually; the first self-ligating bracket was introduced in the early 1930s. They have undergone a revival over the past 30 years with a variety of new appliances being developed. These self-ligating brackets have been touted to possess many advantages over conventional edgewise brackets.

Compared to conventional brackets, the most compelling potential advantages attributed to SLBs are a reduction in overall treatment time [5, 6] and less associated subjective discomfort [7]. Other purported improvements include more efficient chair-side manipulation [8], better infection control [9], and promotion of periodontal health due to proper biohospitality. Preliminary retrospective research had pointed to a definite advantage, with a reduction in overall treatment time of 4 to 7 months and a similar decrease in required appointments [5, 6].

However, whether the levels of OHRQOL in patients were influenced by the type of brackets effects was not clear; thus, our study aims to explore the effect of SLBs on orthodontic treatment with respect to quality of life.

## 2. Materials and Methods

### 2.1. Participants

Ethical approval was obtained from the Ethics Committee of the Stomatology Hospital of the Wenzhou Medical University. The participants were informed about the examination procedures and assured the confidentiality of the collected information. Only those who were given written consent were included in the research.

This was a prospective longitudinal study involving a cohort of patients undergoing fixed orthodontic appliance treatment in the Department of Orthodontics of the Stomatology Hospital, Wenzhou Medical University. A consecutive sample of one hundred fifty patients seeking orthodontic treatment in the department was enrolled in the study. The samples were selected from one profession orthodontist and divided into 2 groups: the self-ligating group with a 0.022∗0.027 inch slot (SL bracket, Damon 3, Ormco, Glendora, Calif) and the conventional bracket group with a 0.022∗0.030 inch slot (CL bracket, 3M Unite, Monrovia, Calif). Both groups consisted of 75 consecutive patients who completed their orthodontic treatment in the Department of Orthodontics. The patients were treated with the same treatment philosophy beginning with the same sequence of 0.012, 0.014, and 0.016 inch and 0.019∗0.025 inch nickel-titanium arch-wires and 0.019∗0.025 inch SS arch-wires in the end.

Exclusion criteria included patients with cognitive disorders, those who had previously received orthodontic treatment, and those with craniofacial anomalies such as cleft lip and palate.

### 2.2. Instruments and Measures

During the interviews, the adolescents provided information concerning demographic factors such as gender, age, and social-economic status before insertion of appliance. For the oral health-related quality of life assessment, the Chinese version of the OHIP-14, which had shown good psychometric properties, was used. The subjects completed five sets of interviews and clinical evaluations at 1 week (T1), 1 month (T2), 3 months (T3), and 6 months (T4) after appliance placement and after treatment (T5).

14 items were included in the Chinese version of the OHIP-14, covering seven conceptualized domains: functional limitation, physical pain, psychological discomfort, physical disability, psychological disability, social disability, and any handicaps. Each item was scored on a 5-point scale to rate the impact of overall oral health status as it relates to the particulars of OHQOL, whereby responses were coded as follows: never (score 0), hardly ever (1), occasionally (2), fairly often (3), and very often (score 4). OHIP-14 scores were calculated by summing the response codes for the 14 items. Consequently, the total scores could range from 0 to 56, with higher scores indicating poorer OHQOL.

### 2.3. Data Analysis

Differences in the distribution of covariates between two groups were tested by using nonparametric tests for repeated measurements and ordinal variables when appropriate and Bonferroni post hoc tests were used to compare between the groups for the quantitative variables measured from T1 to T5. Chi-square tests were used to compare categorical variables.

All analyses were carried out by using Stata software (version 11.2; StataCorp, College Station, Tex). Significance levels were established at 0.05.

## 3. Results

The full study sample comprised 150 young adults (58 men, 92 women) aged 13 to 18 years (mean age of the total sample, 15.6 ± 1.8 years), divided into 2 groups, with each group comprising 75 subjects: self-ligating bracket and conventional bracket. No patients were excluded. The mean treatment time was 21.6 months in self-ligating group and 22.1 months in conventional group.

At baseline, the two groups were comparable with respect to age, gender, dental health component, aesthetic component, and socioeconomic status; see Table 1.

Descriptive statistics indicated no significant differences between the self-ligating group and conventional group for the baseline IOTN-DHC (*P* = 0.783), IOTN-AC (*P* = 0.689), sex (*P* = 0.564), age (*P* = 0.826), and social-economic status (*P* = 0.346).

### 3.1. Total Scores

**Figure 1:**
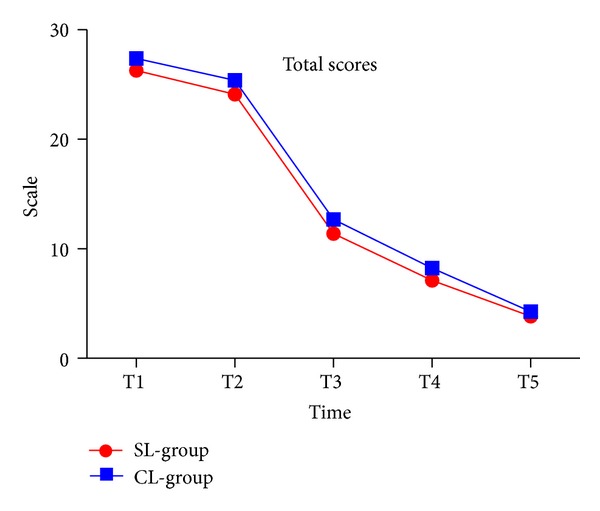
Total score in two groups.

Total scores decreased from T1 to T5 (*P* < 0.001). OHRQOL was consistently lower in the self-ligating group compared to the conventional group, but there were no significant statistical differences between the two groups (Figure 1 and Table 2).

### 3.2. Functional Limitation

**Figure 2:**
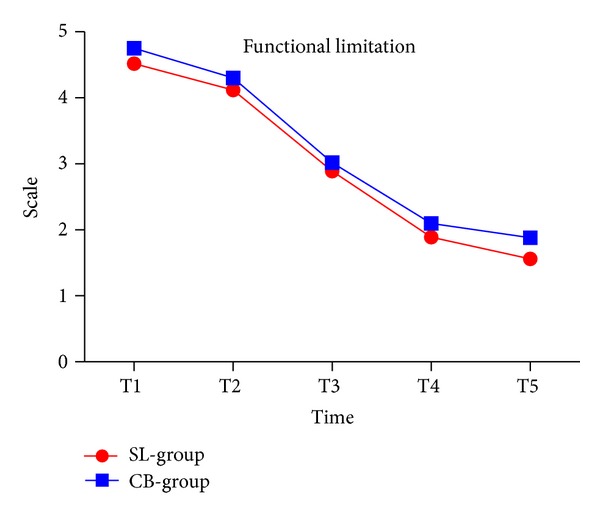
Functional limitation in two groups.

Functional limitation significantly decreased from T1 to T5 (*P* < 0.001) in both groups. Functional limitation was reported to be higher in the conventional group than in self-ligating group at T1 and T2, but there were no significant statistical differences between the two groups (Figure 2 and Table 2).

### 3.3. Physical Pain

**Figure 3:**
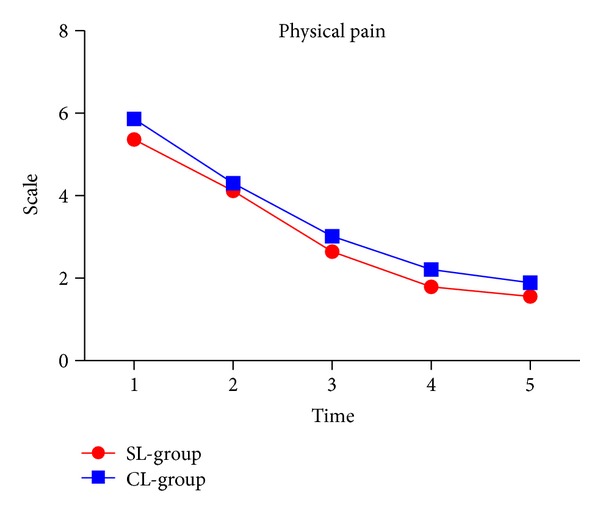
Physical pain in two groups.

We found that bracket type had no effect on pain experience after appliance insertion. However, compared to the self-ligating group, patients reported higher physical pain levels in the conventional group during study period (Figure 3 and Table 2).

### 3.4. Psychological Discomfort

**Figure 4:**
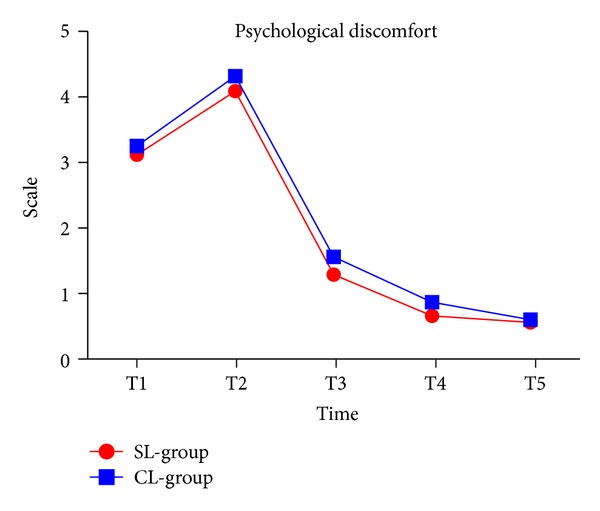
Psychological discomfort in two groups.

Psychological discomfort levels were higher in both groups even though they were not significant. The pattern of psychological discomfort was similar between the two groups. By T3, two groups of patients' psychological discomfort alleviated (Figure 4 and Table 2).

### 3.5. Physical Disability

**Figure 5:**
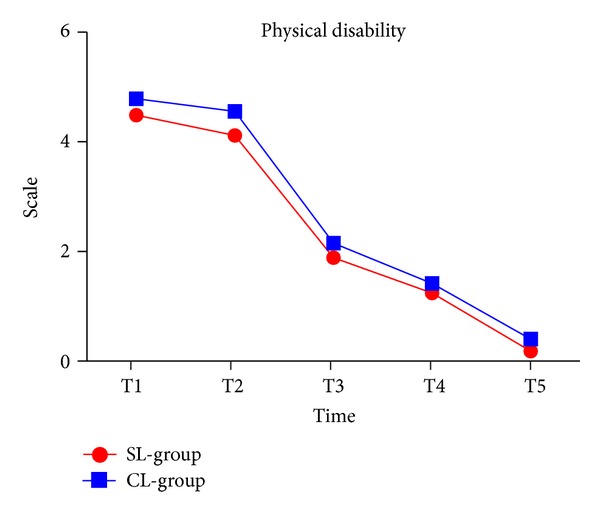
Physical disability in two groups.

Levels of physical disability significantly decreased over time (*P* < 0.001), and there was no significant difference between the groups. Average levels of physical disability were higher in the conventional group than in the self-ligating group (Figure 5 and Table 2).

### 3.6. Psychological Disability

**Figure 6:**
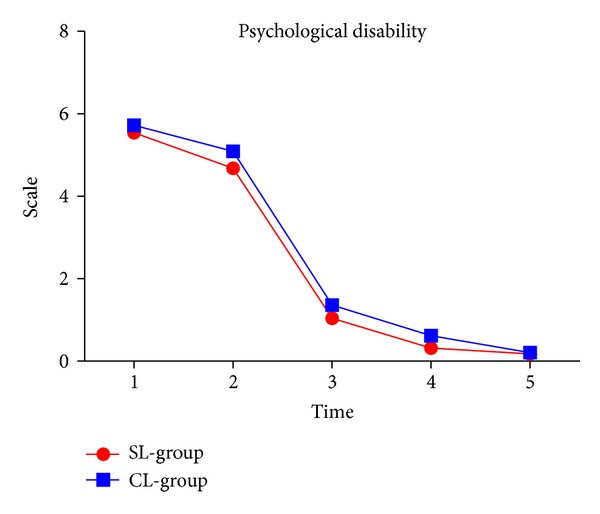
Psychological disability in two groups.

Levels of psychological disability significantly decreased over time (*P* < 0.001). There was no significant difference between the groups. The levels were higher at T1 and T2. However, the levels of psychological disability significantly decreased at T3, T4, and T5 in both groups (Figure 6 and Table 2).

### 3.7. Social Disability

**Figure 7:**
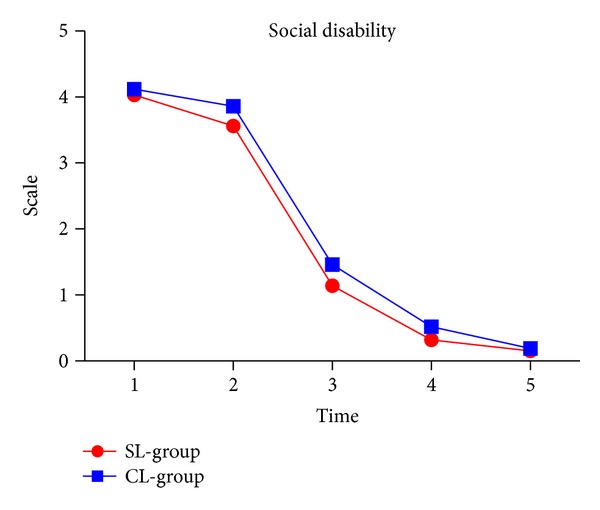
Social disability in two groups.

Levels of social disability significantly decreased at T3 (*P* < 0.001). As time goes by, no matter what kind of brackets is used, it has had little impact on social disability. There was no significant difference between the groups (Figure 7 and Table 2).

### 3.8. Handicaps

**Figure 8:**
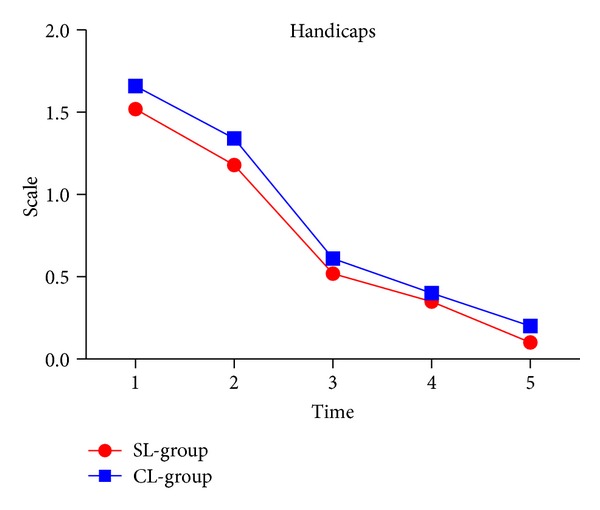
Handicaps in two groups.

Whatever the bracket type is, its impact on handicaps was very little. And there was no significant difference between the groups (Figure 8 and Table 2).

## 4. Discussion

In this study, we conducted a longitudinal prospective study to assess whether two types of orthodontic appliances (self-ligating versus conventional bracket) affected the levels of OHRQOL in Chinese adolescence patients.

At present, the studies of self-ligating brackets focus on traditional aspects, such as peer-reviewed index (peer assessment rating index, PAR), cephalometric changes, and incidence analysis (e.g., treatment of root absorption) compared to conventional brackets. However, the orthodontic treatment, which has a large psychosocial influence, especially for adolescents, calls for the use of OHRQOL measures [10].

The assessment of OHRQOL plays an important role in clinical practice [11]. There are many kinds of evaluation questionnaire to assess OHRQOL in clinic such as CPQ11-14, CSI, and OHIP14. One of the most commonly used generic OHRQOL measures is the two versions of OHIP, with 49 or 14 items, respectively [12]. It is important to carry out a rigorous translation and validation process before an instrument was introduced to another country with different language [13]. Therefore, we applied the Chinese version of the OHIP-14 which showed good reliability and validity [14] to this study.

But questionnaire itself has certain subjectivity, given the fact that patients' age, gender, and economic class will affect the patients' responses. Therefore, this study adopted strict inclusion criteria to guarantee that the patients' baseline was the same, as far as possible to eliminate the bias caused by the inconsistency in patients.

We found that physical pain was higher in the conventional than self-ligating group, although the differences did not yield statistical significance.

Many studies [15–17] found that conventional bracket patients did not suffer from more severe pain during the first week of treatment, compared with self-ligating bracket patients. However, we found the opposite results—Pringle et al. [18] described less perception of pain during self-ligating bracket treatment. The reduction in pain perception may be due to a lighter mechanical force that was applied in the self-ligating bracket in alignment phase, compared with conventional bracket. In both groups, the pain subsided after a week, and the decline was similar in both groups.

Table 2 shows that, one week after the insertion of fixed appliances, the overall OHIP-14 scores and domain scores were the highest of all six different intervals. Primary results focused on physical pain and psychological discomfort, functional limitation, and social disability. Self-ligating brackets group's OHRQOL was superior to the conventional brackets group but the difference was not statistically different. This is because of the orthodontic treatment of pain peaked within one week after the insertion; another reason was that patients were difficult to adapt to wearing appliance while eating or speaking which induce the patient's physical and psychological discomfort. Similar observations have been made by O'Connor [19].

Sergl et al. [20] reported that the most frequent complaints were impaired speaking, impaired swallowing, feeling of oral constraint, and lack of confidence in public after undergoing different appliance treatments. The relation between different types of appliance and the generalized feeling of oral constraint and lack of confidence was not so important.

Existing research [21] shows that these adverse reactions related to bracket size and bonding position. Self-ligating brackets provide a continuous lighter force and efficiency of cleaning because of the small size, so the self-ligating brackets group had better score than conventional group, although there was no statistically significant difference was found.

At 1st month, scores of two groups remained at high levels, but as the treatment continued, OHRQOL scores of the two groups were gradually reduced and no statistical difference was found; this may be due to the improvement in patient's oral function and gradual adaption to the orthodontic treatment. This result suggests that both brackets can treat malocclusion effectively, increase self-confidence, and improve patients OHRQOL level.

We can see from the results that both kinds of brackets will affect patients' OHRQOL, especially physical pain, psychological discomfort, functional limitation, and social disability. This is in contrast to previous claims made by Damon Braces (http://www.damonbraces.com).

But we found that the total OHRQOL scores in the self-ligating group were lower than those of conventional group, which implied that the patients feel more comfortable in self-ligating group, although the difference was not statistically significant. This suggests that, in order to improve the level of OHRQOL, orthodontists can choose self-ligating brackets for patients who were more sensitive to pain or discomfort.

## 5. Limitations and Implications for Future Researches

This study was the first to use valid and reliable instruments to investigate the effects of self-ligating and conventional brackets on the oral health-related quality of life of young patients who had completed treatment. The subjects in this study were reasonably comparable, since there were no significant differences between them at baseline with regard to the dental health component, sex, age, and socioeconomic class. Nevertheless, some limitations should be addressed. First, because of the small size of the samples, the results might not be generalizable to the Chinese adolescents. Second, strict inclusion criteria and same baseline were adopted, but the reflection of everyone to the pain and discomfort may be different, so there is still another objective bias will affect the final result. Finally, a randomized controlled trial is the highest level of evidence, but it is hard to conduct a random trial for ethical reasons.

In the future, large sample and randomized research should be carried out which will provide a more accurate profile of bracket type' impact on oral health-related quality of life. Moreover, patients' responses caused bias. However, this limitation is present in other studies [22], and further research is necessary to eliminate the effect of the bias for the results.

## 6. Conclusion

1. Use of self-ligating bracket did not improve overall level of OHRQOL when compared with conventional ligated brackets.
2. Average levels of OHRQOL were higher in the conventional group than in the self-ligating group.

## Figures and Tables

**Table 1:** Frequency distribution of self-ligating and conventional subjects' characteristics.

	Total		Self-ligating		Conventional		*P* value (chi-square test)
	%	(*n*)	%	(*n*)	%	(*n*)	
Total	100	150	50	75	50	75	
Baseline IOTN-DHC							
No or little need	100	6	50	3	50	3	0.783
Borderline need	100	22	54	12	46	10	
Need	100	122	53	65	47	57	
Sex							
Female	100	92	52	48	48	44	0.564
Male	100	58	50	29	50	29	
Baseline IOTN-AC (normative)							
No or little need	100	4	50	2	50	2	0.689
Borderline need	100	20	55	11	45	9	
Need	100	126	56	70	44	56	
Age (y)							
13–15	100	48	46	22	54	26	0.826
16–18	100	102	51	52	49	50	
Social-economic class							
(high)	100	134	56	75	44	59	0.346
(low)	100	12	42	5	58	7	

IOTN: index of orthodontic treatment need; DHC: dental health component; AC: aesthetic component.

**Table 2:** Means (and standard deviations) of functional limitation, physical pain, psychological discomfort, physical disability, psychological disability, social disability, and any handicaps in the two types of orthodontic appliances from T1 to T5.

Appliance	Time	Functional limitation	Physical pain	Psychological discomfort	Physical disability	Psychological disability	Social disability	Handicaps	Total scores
Self-ligating bracket	T1	4.52 (1.89)	5.36 (1.65)	3.12 (1.12)	4.49 (1.64)	5.54 (1.50)	4.03 (1.20)	1.52 (0.86)	26.32 (2.35)
Conventional bracket		4.75 (1.99)	5.86 (1.81)	3.25 (1.09)	4.79 (1.72)	5.72 (1.44)	4.12 (1.10)	1.66 (0.81)	27.40 (2.15)

Self-ligating bracket	T2	4.12 (1.78)	4.12 (1.85)	4.09 (1.42)	4.12 (1.73)	4.68 (1.36)	3.56 (0.98)	1.18 (0.89)	24.12 (2.16)
Conventional bracket		4.30 (1.56)	4.30 (1.89)	4.32 (1.23)	4.56 (1.64)	5.09 (1.42)	3.86 (0.88)	1.34 (0.85)	25.46 (2.14)

Self-ligating bracket	T3	2.89 (1.14)	2.64 (1.34)	1.29 (0.92)	1.89 (0.82)	1.04 (0.98)	1.14 (0.78)	0.52 (0.75)	11.47 (2.03)
Conventional bracket		3.02 (1.23)	3.02 (1.46)	1.56 (0.85)	2.15 (0.75)	1.36 (0.86)	1.46 (0.69)	0.61 (0.72)	12.76 (2.31)

Self-ligating bracket	T4	1.89 (0.76)	1.79 (0.97)	0.66 (0.70)	1.24 (0.42)	0.32 (0.29)	0.32 (0.45)	0.35 (0.16)	7.12 (1.89)
Conventional bracket		2.10 (0.88)	2.21 (1.02)	0.87 (0.69)	1.42 (0.35)	0.62 (0.38)	0.52 (0.52)	0.40 (0.22)	8.23 (1.74)

Self-ligating bracket	T5	1.56 (0.70)	1.56 (0.71)	0.56 (0.18)	0.18 (0.14)	0.18 (0.13)	0.15 (0.21)	0.10 (0.19)	3.85 (0.59)
Conventional bracket		1.88 (0.68)	1.89 (0.98)	0.60 (0.21)	0.40 (0.11)	0.21 (0.12)	0.19 (0.11)	0.20 (0.13)	4.25 (0.49)

## References

[1] Coulter ID, Marcus M, Atchison KA (1994). Measuring oral health status: theoretical and methodological challenges. *Social Science and Medicine*.

[2] Engel GL (2012). The need for a new medical model: a challenge for biomedicine. *Psychodynamic Psychiatry*.

[3] Kiyak HA (2008). Does orthodontic treatment affect patients’ quality of life?. *Journal of Dental Education*.

[4] Inglehart M, Bagramian R (2002). *Oral Health Related Quality of Life*.

[5] Harradine NW (2001). Self-ligating brackets and treatment efficiency. *Clinical Orthodontics and Research*.

[6] Eberting JJ, Straja SR, Tuncay OC (2001). Treatment time, outcome, and patient satisfaction comparisons of Damonand conventional brackets. *Clinical Orthodontics and Research*.

[7] Damon DH (1998). The Damon low-friction bracket: a biologically compatible straight-wire system. *Journal of Clinical Orthodontics*.

[8] Turnbull NR, Birnie DJ (2007). Treatment efficiency of conventional vs self-ligating brackets: effects of archwire size and material. *American Journal of Orthodontics and Dentofacial Orthopedics*.

[9] Maijer R, Smith DC (1990). Time savings with self-ligating brackets. *Journal of Clinical Orthodontics*.

[10] De Oliveira CM, Sheiham A (2004). Orthodontic treatment and its impact on oral health-related quality of life in Brazilian adolescents. *Journal of Orthodontics*.

[11] Locker D, Matear D, Stephens M, Lawrence H, Fayne B (2001). Comparison of the GOHAI and OHIP-14 as measures of the oral health-related quality of life of the elderly. *Community Dentistry and Oral Epidemiology*.

[12] Bernabé E, de Oliveira CM, Sheiham A (2008). Comparison of the discriminative ability of a generic and a condition-specific OHRQoL measure in adolescents with and without normative need for orthodontic treatment. *Health and Quality of Life Outcomes*.

[13] Chen M, Wang D, Wu L (2010). Fixed orthodontic appliance therapy and its impact on oral health-related quality of life in Chinese patients. *Angle Orthodontist*.

[14] Wong MCM, Lo ECM, McMillan AS (2002). Validation of a Chinese version of the Oral Health Impact Profile (OHIP). *Community Dentistry and Oral Epidemiology*.

[15] Scott P, Sherriff M, DiBiase AT, Cobourne MT (2008). Perception of discomfort during initial orthodontic tooth alignment using a self-ligating or conventional bracket system: a randomized clinical trial. *European Journal of Orthodontics*.

[16] Fleming PS, DiBiase AT, Sarri G, Lee RT (2009). Pain experience during initial alignment with a self-ligating and a conventional fixed orthodontic appliance system. *Angle Orthodontist*.

[17] Miles P, Weyant R (2010). Porcelain brackets during initial alignment: are self-ligating cosmetic brackets more efficient?. *Australian Orthodontic Journal*.

[18] Pringle AM, Petrie A, Cunningham SJ, McKnight M (2009). Prospective randomized clinical trial to compare pain levels associated with 2 orthodontic fixed bracket systems. *American Journal of Orthodontics and Dentofacial Orthopedics*.

[19] O’Connor PJ (2000). Patients’ perception before, during, and after orthodontic treatment. *Journal of Clinical Orthodontics*.

[20] Sergl HG, Klages U, Zentner A (1998). Pain and discomfort during orthodontic treatment: causative factors and effects on compliance. *American Journal of Orthodontics and Dentofacial*.

[21] Mandall NA, Matthew S, Fox D (2008). Prediction of compliance and completion of orthodontic treatment:are quality of life measure important?. *European Journal of Orthodontics*.

[22] Liu Z, McGrath C, Hagg U (2009). The impact of malocclusion/orthodontic treatment need on the quality of life a systematic review. *Angle Orthodontist*.

